# Outcomes of Delayed Norwood Operation in High-Risk Patients—Time to Reconsider the Dogma of a Primary Norwood Operation?

**DOI:** 10.1093/icvts/ivag218

**Published:** 2026-08-28

**Authors:** Mahmut Ozturk, Arif Selcuk, Aybala Tongut, Elizabeth M Moynihan, Manan Desai, Alejandro Jose Lopez Magallon, Mary T Donofrio, Heather Gordish-Dressman, Kathleen Kalinger Reid, Ricardo Alfonso Munoz, Yves d’Udekem, Gil Wernovsky, Can Yerebakan

**Affiliations:** Department of Cardiac Surgery, Children’s National Hospital, The George Washington University School of Medicine and Health Sciences, Washington, DC 20010, United States; Department of Cardiac Surgery, Children’s National Hospital, The George Washington University School of Medicine and Health Sciences, Washington, DC 20010, United States; Department of Cardiac Surgery, Children’s National Hospital, The George Washington University School of Medicine and Health Sciences, Washington, DC 20010, United States; Department of Cardiac Surgery, Children’s National Hospital, The George Washington University School of Medicine and Health Sciences, Washington, DC 20010, United States; Department of Cardiac Surgery, Children’s National Hospital, The George Washington University School of Medicine and Health Sciences, Washington, DC 20010, United States; Department of Cardiac Critical Care Medicine, Children’s National Hospital, The George Washington University School of Medicine and Health Sciences, Washington, DC 20010, United States; Department of Cardiology, Children’s National Hospital, The George Washington University School of Medicine and Health Sciences, Washington, DC 20010, United States; Research Center for Genetic Medicine, Children’s National Hospital, Washington, DC, United States; Department of Cardiac Critical Care Medicine, Children’s National Hospital, The George Washington University School of Medicine and Health Sciences, Washington, DC 20010, United States; Department of Cardiac Critical Care Medicine, Children’s National Hospital, The George Washington University School of Medicine and Health Sciences, Washington, DC 20010, United States; Department of Cardiac Surgery, Children’s National Hospital, The George Washington University School of Medicine and Health Sciences, Washington, DC 20010, United States; Department of Cardiac Critical Care Medicine, Children’s National Hospital, The George Washington University School of Medicine and Health Sciences, Washington, DC 20010, United States; Department of Cardiology, Children’s National Hospital, The George Washington University School of Medicine and Health Sciences, Washington, DC 20010, United States; Department of Cardiac Surgery, Children’s National Hospital, The George Washington University School of Medicine and Health Sciences, Washington, DC 20010, United States

**Keywords:** hypoplastic left heart, hybrid palliation, Norwood operation, bilateral pulmonary artery banding

## Abstract

**Objectives:**

The delayed Norwood (DN) approach, preceded by bilateral pulmonary artery banding (bPAB), has emerged as a strategy to improve outcomes in high-risk infants by addressing neonatal haemodynamic instability and organ immaturity.

**Methods:**

This single-centre, retrospective study analysed 20 consecutive patients with hypoplastic left heart syndrome/variants and multiple risk factors who underwent a DN operation following bPAB between 2019 and 2024.

**Results:**

Bilateral pulmonary artery banding was performed at a median age of 2.5 days (interquartile range [IQR]: 2-4). Preoperative risk factors included prematurity (35%), low birth weight <2.5 kg (25%), non-cardiac comorbidities (25%), genetic syndromes (50%), preoperative cardiogenic shock (35%), and restrictive atrial septum (25%). The median age at DN was 36 days (IQR: 29-79), with an operative survival rate of 75%. Patients who underwent the DN within 4 weeks of bPAB did not require any pulmonary artery (PA) reinterventions. Median lactate clearance time was 8 hours (IQR: 5-18), and the median vasoactive inotropic score decreased by 47% within 48 hours postoperatively. Cerebral near-infrared spectroscopy values improved in patients without postoperative extracorporeal membrane oxygenation (ECMO) support from 53% to 58% and 63% at 0, 12, and 36 hours, respectively.

**Conclusions:**

The DN approach, preceded by initial bPAB in a high-risk patient cohort, achieved surgical outcomes comparable to those of lower-risk patients undergoing primary Norwood operation. A DN procedure around 4-6 weeks of following bPAB may improve survival rates in high-risk patients with a reduction in surgical or catheter-based reinterventions.

## Graphical abstract

**Figure:**
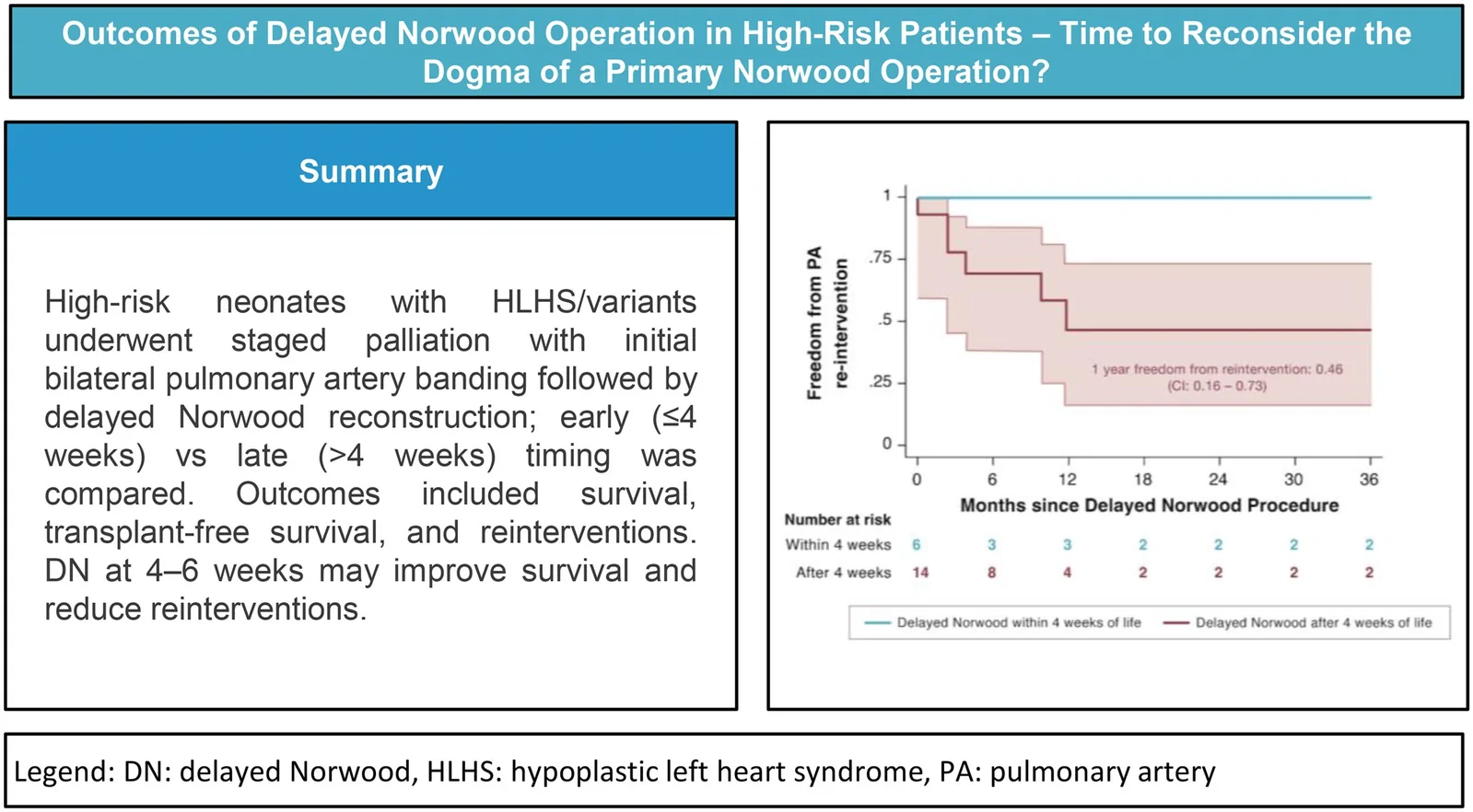

## INTRODUCTION

Over 40 years have passed since the seminal report of Norwood, Lang, and Hansen describing the first successful surgical palliation of hypoplastic left heart syndrome (HLHS).^1^ The child described in the case report lived well into his 20s, and today even the smallest neonates with HLHS can be palliated well into infancy.^2^ However, despite ingenious modifications of the surgical technique and advances in the postoperative management, survival rates of patients with HLHS are far from expectations.^3^^,^^4^ The landmark Single Ventricle Reconstruction Trial suggested that the Sano modification fared better over the short term, and particularly given its longitudinal nature, the report emphasized the need for improvement in all aspects of care, particularly unplanned interventions, days in hospital, and the duration and quality of life.^5–8^

While mostly spared for high-risk patients in the United States,^9^ specialized institutions have utilized the hybrid approach with bilateral pulmonary artery banding (bPAB) and ductal stenting for all-comers with HLHS reporting excellent short and long-term outcomes.^10^^,^^11^ Recent reports from the Society of Thoracic Surgeons database revealed satisfactory outcomes for the hybrid palliation compared to the Norwood procedure despite being spared mostly for high-risk patients and despite the lack of standardization of the hybrid procedure and it postoperative/interstage management.^12^

Recently, we proposed a developmentally based approach to the neonate with HLHS.^13^ Recognizing the 3 most vulnerable periods in the first year of life to be (1) the transitional circulation, (2) the early postoperative period after the Norwood procedure, and (3) the “interstage” period prior to the bidirectional Glenn (BDG), we suggested (1) rapid control of pulmonary blood flow by bPAB within 24-48 hours of age, (2) deferring the Norwood period until there is improved organ maturity, neuro-developmental care and full oral feeding, usually at ∼3-4 weeks of age, “rapid two-stage Norwood strategy” (RTSN), and (3) shortening the interstage period until the BDG.

Since 2017, our programme has been in a transition where we utilize all 3 approaches where appropriate. The purpose of this report is to describe (1) the hospital course before the delayed Norwood (DN) procedure, (2) the intraoperative and early postoperative course after the DN procedure, (3) the hospital course and events prior to the BDG, and (4) mid-term follow-up of patients who underwent a DN following bPAB. This report will also include the lessons we learned and proposals regarding future strategies for the surgical treatment of patients with HLHS.

## METHODS

**Figure 1.**
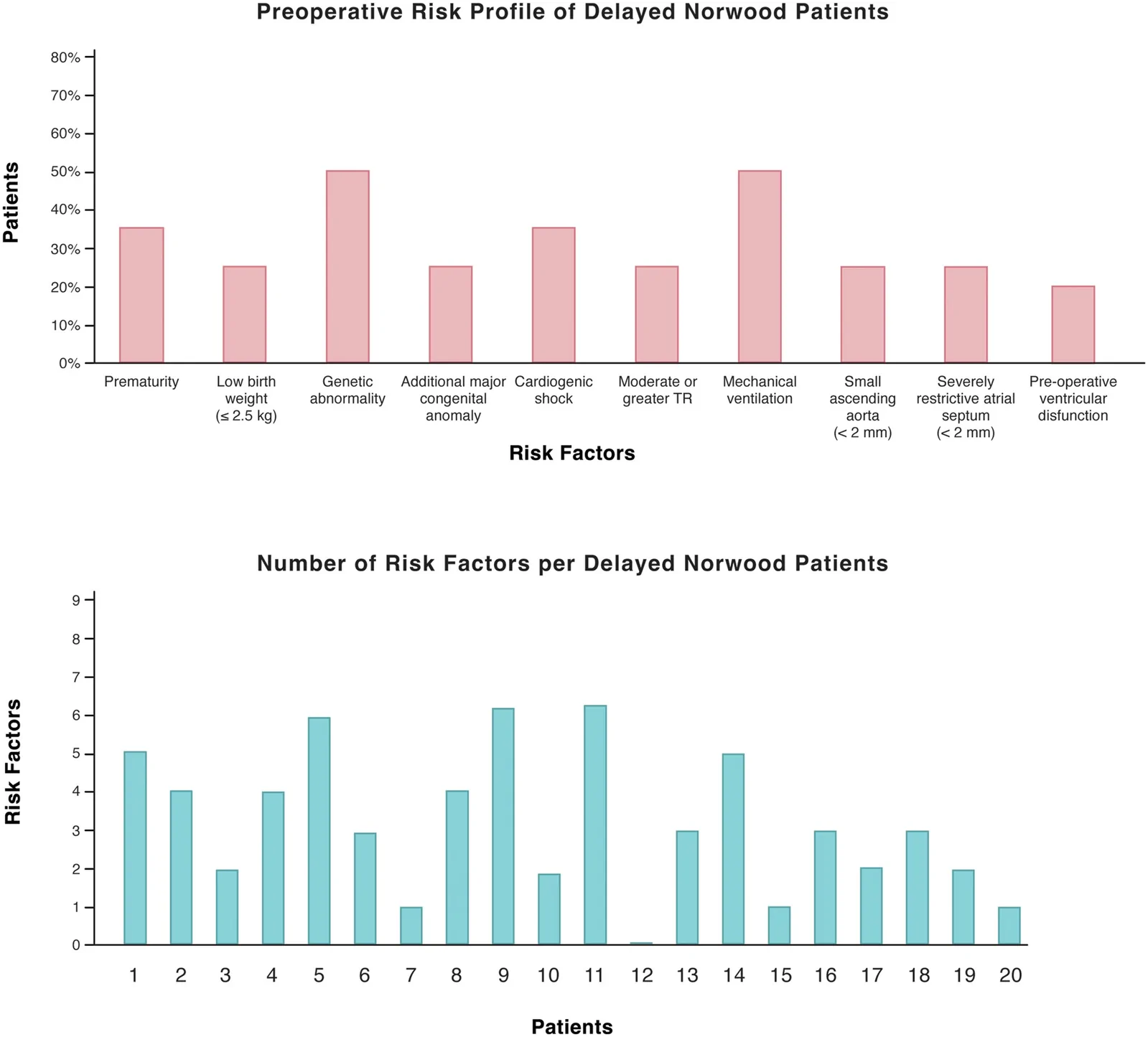
Preoperative Risk Factors in Delayed Norwood Patients Prior to Bilateral Pulmonary Artery Banding. Abbreviation: TR, tricuspid regurgitation

A single-centre retrospective review of consecutive patients with HLHS, HLHS variants, or other single ventricle lesions who underwent DN palliation from January 30, 2019, to February 8, 2024, was approved by the Institutional Review Board at Children’s National Hospital (Pro00015566; 07/01/2021), with a waiver of consent. Patients who died or underwent heart transplantation before DN, or who underwent hybrid palliation with subsequent comprehensive stage 2, were excluded. All available data up to April 30, 2024, were obtained retrospectively from institutional records. Definitions are in **Supplemental Material**. Patient characteristics are shown in **Table 1**, and institutional high-risk criteria are summarized in **Figure 1**. Given extracorporeal membrane oxygenation (ECMO)’s potential impact on postoperative parameters, only patients without ECMO were included in analyses of early postoperative vasoactive-inotropic score (VIS), lactate, and urine output.

**Table 1.** Patient Demographics

Variable		*n* = 20
Male (%)		9 (45)
Weight at birth, kg		2.8 (2.5-3.4)
<2.5 kg birth weight (%)		5 (25)
Prenatal diagnosis (%)		18 (90)
Additional major congenital anomaly (%)		5 (25)
Heterotaxy syndrome		3 (15)
Turners syndrome		1 (5)
Bardet-Biedl syndrome		1 (5)
Premature (<37 weeks) (%)		7 (35)
Identified genetic abnormality (%)		10 (50)
Echocardiography (%) or median (IQR 25-75)	Single RV/HLHS	7/12 (58)
	Ascending aorta z-score	−3.7 (−4.2 to −2.0)
	Endocardial fibroelastosis	2 (10)
	Restrictive/intact interatrial septum at admission	5 (25)
	TAPVR	1 (5)
	Coronary-cameral fistula	4 (20)
	≥Moderate TR	5 (25)
Prior to first surgery		Pre-bPAB
Age at first surgery, days		2.5 (2-4)
Need for BAS (%)		7 (35)
Highest serum lactate, mmol/L		3.5 (2.8-5.1)
Mechanical ventilation (%)		10 (50)
Highest VIS		0 (0-2.5)
GFR on day of first surgery		28 (24-33)
Shock/metabolic acidosis (%)		7 (35)
Ever PO feeding (at any time) (%)		2 (10)
Ever PO + tube feeding (%)		2 (10)
NPO (%)		18 (90)
NEC (%)		1 (5)
Sepsis (%)		1 (5)

Values are represented as median (interquartile range Q1-Q3) or *n* (%).

Abbreviations: BAS, balloon atrial septostomy; bPAB, bilateral pulmonary artery banding; GFR, estimated glomerular filtration rate; HLHS, hypoplastic left heart syndrome; kg, kilograms; mg/dL, milligrams per decilitre; mmol/L, millimoles per litre; NEC, necrotizing enterocolitis; NPO, nothing by mouth; PO, per oral; RV, right ventricle; TAPVR, total anomaly of pulmonary venous return; TR, tricuspid regurgitation; VIS, vasoactive inotropic score.

The bPAB and hybrid stage 1 palliation were performed as previously described.^10^ The DN surgery was performed as seen in the surgical video (**Supplemental Material, Video 1**). The choice of Sano versus Blalock-Taussig shunts was based on surgeon preference and evolving institutional experience, with increasing use of the Sano shunt during the study period. This change was based on our increasing clinical impression that the Sano shunt was associated with more favourable pulmonary artery (PA) physiology, perhaps due to its more pulsatile antegrade flow pattern and the fact that it did not require a distal anastomosis on previously banded, and therefore potentially fragile, segments of the PA. Furthermore, postoperative ICU management seemed to be easier for patients with a Sano shunt configuration, which further encouraged its growing use in this high-risk DN population.

Outcomes of interest included interstage clinical and haemodynamic data from bPAB to DN, as well as operative, early postoperative, and mid-term outcomes following DN, with particular focus on surgical and catheter-based reinterventions and stratification according to the timing of the DN procedure.

### Statistical analysis

Categorical variables are reported as frequencies and percentages; continuous variables are presented as medians with interquartile ranges (IQRs) due to non-Gaussian distribution. Continuous variables were tested for normality using the Shapiro-Wilk test. Group comparisons were performed using Student’s t-test or Mann-Whitney *U*-test for continuous variables, and chi-squared or Fisher’s Exact test for categorical variables, as appropriate (**Table E1**). Kaplan-Meier curves estimated overall survival and time to PA reintervention function, compared via log-rank tests. For the freedom-from-reintervention analysis, death was considered a censored event rather than a competing risk due to the small number of events in the cohort. Statistical significance was set at *P* < .05. Data analysis was conducted using RStudio version 4.3.1.

## RESULTS

A total of 48 mostly high-risk patients underwent initial bPAB during the study period. Overall 5 of these (10.4%) died following bPAB. Of these, 2 deaths occurred under compassionate care as elected by the parents. Among the remaining 3, only 2 were in-hospital mortalities—1 due to hypoxic brain injury secondary to bradycardia, and 1 due to necrotizing enterocolitis. One patient experienced sudden cardiopulmonary deterioration and died at home during the interstage period. In one case, bPAB served as a bridge to heart transplantation due to severe tricuspid regurgitation.

Twenty patients underwent DN at a median age of 36 days (IQR: 29-79). The patient cohort included 5 patients with aortic atresia and mitral stenosis, 6 with aortic atresia and mitral atresia, 1 with aortic stenosis and mitral stenosis, 3 with mitral atresia and double outlet right ventricle (DORV), 1 with mitral atresia, DORV, and ventricular septal defect, 1 with mitral atresia, DORV, and subaortic stenosis, 1 with an unbalanced atrioventricular canal defect (right dominant) and aortic valve atresia, 1 with an unbalanced atrioventricular canal defect (right dominant) associated with subaortic stenosis, small aortic annulus, hypoplastic aortic arch, and left isomerism, and 1 patient with tricuspid atresia and malposition of the great arteries. Of the 20 patients, 6 patients (30%) underwent DN procedure within 4 weeks and 14 (70%) underwent DN procedure beyond 4 weeks following bPAB (**Table E1**).

Out of 20 patients, 8 (40%) patients who underwent ductal stent placement following bPAB were initially expected to wait for a comprehensive stage 2 procedure. However, due to unfavourable clinical and haemodynamic circumstances—such as retrograde arch obstruction, ductal stent obstruction, inability to separate from non-invasive respiratory support, and atrioventricular valve insufficiency—these 8 patients underwent a DN procedure. Data following bPAB until DN are summarized in **Table E2**.

### Preoperative patient characteristics and intraoperative variables

The preoperative risk factors of the DN patients prior to bPAB banding were genetic abnormality (*n* = 10, 50%), preoperative mechanical ventilation (*n* = 10, 50%), cardiogenic shock (*n* = 7, 35%), and prematurity (*n* = 7, 35%). The median (range) number of risk factors per patient was 3 (0-6). Sixty percent of the patients (12/20) had 3 or more risk factors prior to bPAB. A balloon atrial septostomy prior to surgery was required in 5 (25%) patients (**Figure 1**).

At the time of DN procedure, the median weight was 3.5 kg (IQR: 3.2-3.9). Additionally, estimated GFR was 88 mL/min/1.73 m^2^ (IQR: 62-95) and the VIS was zero (excluding the single patient with preoperative ECMO support). Seven of the patients (35%) required mechanical ventilation at the time of surgery and 5 (25%) were on oral diets before surgery.

During the DN operation, the total bypass and total cross-clamp times were 238 (IQR: 219-280) and 85 (IQR: 66-98) min. Hypothermia was established at 28 (26-28) degrees. Eight (40%) of the patients received a Sano shunt and 11 (55%) patients received a modified Blalock-Taussig shunt. In one patient central shunt was placed for pulmonary blood flow. For the arch reconstruction femoral vein homograft was used in 3 (15%), PA homograft in 8 (40%) and bovine pericardial xenograft in 9 (45%). Operative characteristics are listed on the **Table 2**.

**Table 2.** Norwood Operative Data

Variables	*n* = 20
GFR^a^	88 (62-95)
Mechanical ventilation^a^	7 (35)
VIS^a^	0 (0-0)
Total bypass time (minutes)	238 (219-289)
Total cross-clamp time (minutes)	85 (65-98)
Total RCP time (minutes)	53 (32-70)
DHCA	0
Hypothermia, °C	28 (26-28)
Lower body perfusion utilized	9 (45)
Sano modification	8 (40)
Blalock-Taussig-Thomas shunt	11 (55)
Arch reconstruction—femoral vein homograft	3 (15)
Arch reconstruction—bovine pericardial patch (CardioCel)^19^	9 (45)
Arch reconstruction—pulmonary homograft	8 (40)
Atrial septectomy (y, %)	20 (100)
Additional runs CPB (%)	6 (30)
Delayed sternal closure (%)	20 (100)
VIS leaving OR^b^	6.5 (1-18)
Lactate leaving OR^b^	4.3 (1.6-3.8)
Inhaled NO	12 (60)

Values are represented as median (interquartile range Q1-Q3) or *n* (%).

a Morning of surgery.

b Values excluding the patients with ECMO support.

Abbreviations: °C: centigrade degrees; DHCA, deep circulatory arrest; GFR, glomerular filtration rate; min, minutes; NO, nitric oxide; OR, operating room; RCP, retrograde cerebral perfusion; VIS, vasoactive inotropic score.

### Early postoperative outcomes

One patient left the operating room with ECMO support. Of the remaining 19, 7 (37%) other patients also needed an ECMO support within the first week following DN procedure. Extracorporeal membrane oxygenation weaning was successful in 7 of the 8 patients (88%), and 6 of these 8 (75%) patients could be discharged home.

**Figure 2.**
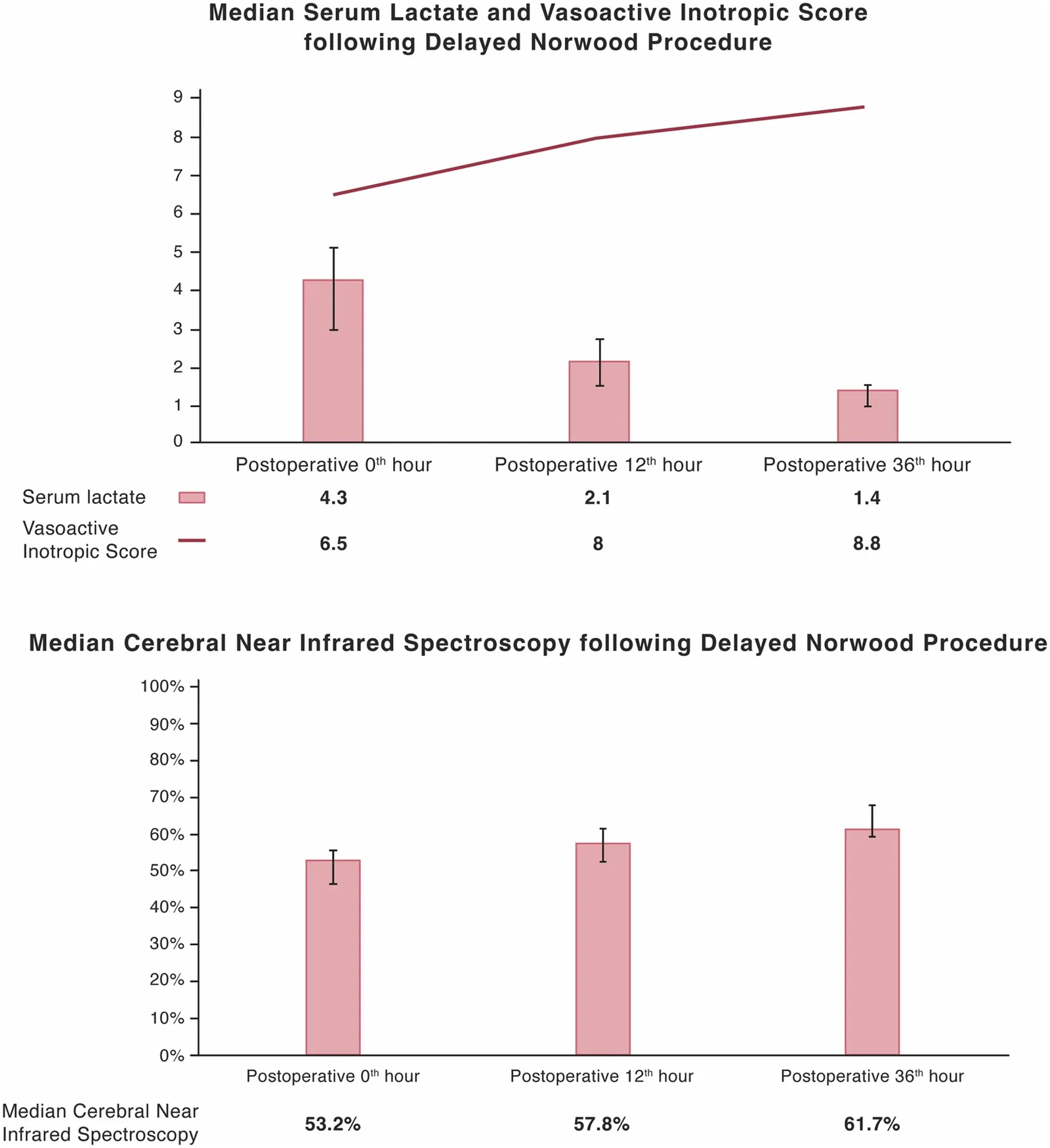
Median Serum Lactate, Vasoactive Inotropic Score, and Cerebral Near-Infrared Spectroscopy in Primary Norwood and Delayed Norwood at 0th, 12th, and 36th Postoperatively

The median lactate at the 0th postoperative hour was 4.3 (IQR: 1.6-11.8) mmol/L, VIS was 6.5 (IQR: 1-18) and near-infrared spectroscopy (NIRS) was 53.2% (IQR: 40.6-65.3). The postoperative serum lactate levels, VIS, and NIRS at 0, 12, and 36 hours are presented **Figure 2**.

The median peak serum lactate levels within the initial 48 hours following the Norwood procedure were 2.24 (IQR: 0-12.5) mmol/L. The median time to normalization of serum lactate (<2 mmol/L) after the DN procedure was 8 hours (IQR: 5-18). The median maximal immediate postoperative vasoactive inotropic score (15 (IQR: 13-18)) was reduced by 47% (8 (IQR: 8-9)) within the first 48 hours following surgery. The median estimated GFR was 61 mL/min/1.73 m^2^ (IQR: 48-70) on the postoperative day 2 after DN. The daily medians for maximum VIS, lactate, NIRS, urine output, GFR as well as the number of patients on ECMO and discharge after ECMO is presented in **Table E3**.

Delayed sternal closure was performed in 19 (95%) patients at a median of 4 days (IQR: 1-6). Median total mechanical ventilation time was 13 days (IQR: 4-31), and reintubations at any time were needed in 15 (75%) patients. The use of a gastrostomy tube was needed in 11 (73%). Median time to achieve full enteral feeding was 14 days (IQR: 12-22). Postoperative length of cardiac intensive care unit (CICU) and hospital stays for in-hospital survivors after the Norwood procedure were 41 (IQR: 23-128) and 54 (IQR: 43-156), respectively (**Table 3**).

**Table 3.** Overall Hospital Course

Variables	*n* = 20
Hospital survivors	15 (75)
EEG seizures	8 (40)
Total opioid received, mg/kg	47 (19-192)
Mechanical ventilation, d	13 (4-31)
Acute kidney injury	9 (45)
Necrotizing enterocolitis	1 (5)
Reintubation	15 (75)
Cardiopulmonary resuscitation	7 (35)
Postoperative ECMO within 7 days	7 (35)
Total ECMO time, hours	117 (72-160)
SVT requiring treatment	2 (10)
VT requiring treatment	2 (10)
Re-exploration for bleeding	3 (15)
Days (range) sternum open	4 (1-6)
Diaphragm plication	1 (5)
Vocal cord paralysis (received injection therapy)	2 (10)
Pleural effusion requiring drainage	0
Bacteraemia	3 (15)
Postoperative length of stay in CICU for in-hospital survivors after Norwood procedure; days	41 (23-128)
Postoperative length of hospital stays for in-hospital survivors after Norwood procedure; days	54 (43-156)
Total days out of hospital within the first year after Norwood procedure for in-hospital survivors	204 (138-303)

Values are represented as median (interquartile range Q1-Q3) or *n* (%).

Abbreviations: CICU, cardiac intensive care unit; ECMO, extracorporeal membrane oxygenation; SVT, supraventricular tachycardia; VT, ventricular tachycardia.

### Survival

**Figure 3.**
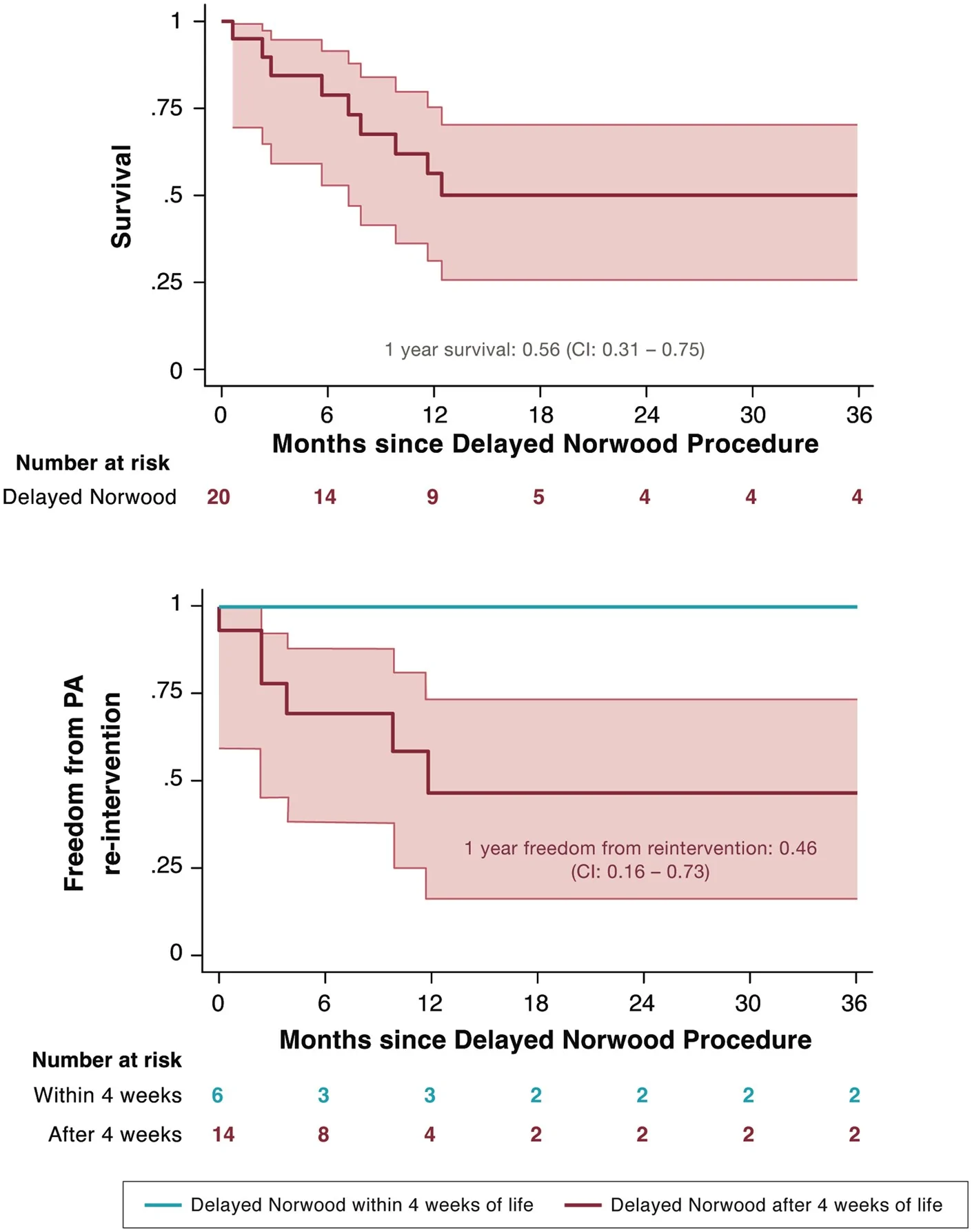
Kaplan-Meier Estimates for Pulmonary Artery Reintervention in Delayed Norwood Patients Who Underwent the Surgery Within or Beyond the Fourth Week of Bilateral Pulmonary Artery Banding (Bottom) and for Survival in Delayed Norwood Patients (Top). Abbreviations: CI, confidence interval; PA, pulmonary artery

Thirty-day survival following the DN procedure was 95%. There were 5 (25%) in-hospital deaths after the DN; 2 with global neurologic injuries following e-CPR (1 patient with tracheal stenosis), 2 with sepsis, and 1 with persistent low cardiac output syndrome and airway complications. In-hospital mortality data of patients who underwent DN were presented in **Table E4.** The Kaplan-Meier estimate for 1-year-survival was 56%. (0.56; CI: 0.31-0.75) (**Figure 3**).

### Reinterventions/reoperations

Eleven (55%) patients needed unplanned (surgical or transcatheter) reinterventions. A total of 9 (45%) patients needed therapeutic catheter-based reintervention within a median of 79 (IQR: 45-97) days. Of these, 6 (30%) were in-hospital reinterventions. The most common catheter-based reintervention was shunt reinterventions (*n* = 5, 25%). This was followed by aortic arch reinterventions in 3 patients (15%), and PA reinterventions in 3 patients (15%). Pulmonary artery reoperation or catheter-based reintervention were performed in 7 (35%) patients, all of which underwent DN operation beyond the fourth week after the initial bPAB. Those who received DN within the first 4 weeks of bPAB did not require PA interventions. The Kaplan-Meier estimate for freedom from reintervention for patients who underwent bPAB beyond 4 weeks of life was 46% (0.46; CI: 0.16-0.73) (**Figure 3**).

Five patients (25%) underwent an unplanned reoperation within a median of 2 days (IQR: 1-15): 5 shunt revisions including 4 PA plasties and 1 arch re-reconstruction. Data on unplanned catheter-based reinterventions or reoperations are provided in **Table E5**.

**Figure 4.**
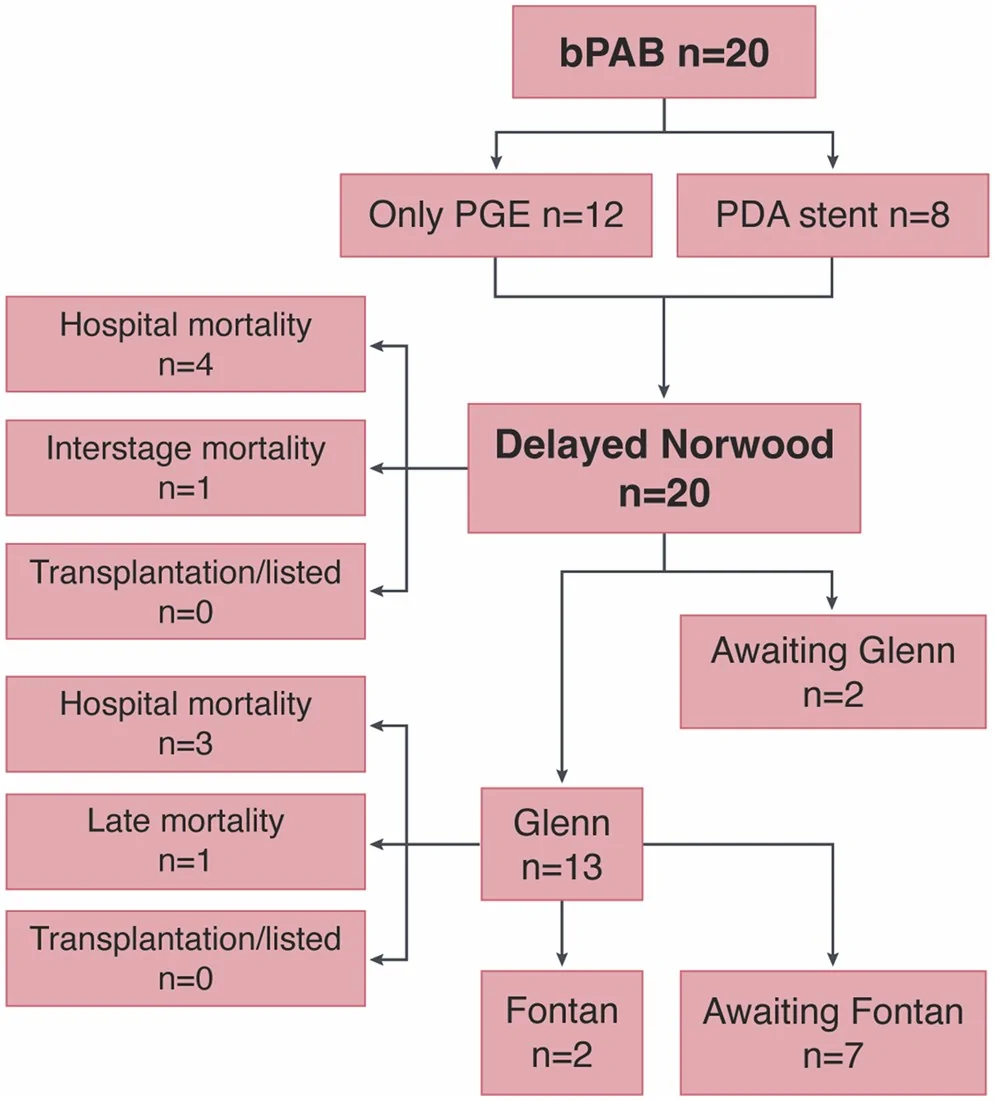
Flowchart of Delayed Norwood Patients. Abbreviation: bPAB, bilateral pulmonary artery banding. Abbreviation: PDA, Patent Ductus Arteriosus; PGE, Prostaglandin

Thirteen (65%) patients underwent a BDG anastomosis at 5.8 months (IQR: 4.9-6.1) (**Table E6**). Fontan circulation completion was achieved in 4 patients (20%); 7 patients (35%) were awaiting the Fontan procedure. None of the patients required heart transplantation (**Figure 4**). The Kaplan-Meier estimate for 1-year freedom from PA reinterventions was 61% (0.61; CI: 0.31-0.81).

## COMMENTS

Hybrid palliation with bPAB, initially introduced as an alternative to the Norwood operation for the management of HLHS and its variants, has demonstrated outcomes comparable to the Norwood procedure when utilized even in high-risk patients and also in all comers in select specialized centres.^9–11^^,^^14^ As previously proposed, this approach is increasingly recognized not only as an exclusive alternative but as a scientifically complementary strategy that can be effectively integrated with the Norwood operation, offering a synergistic pathway to optimize outcomes and potentially improve long-term survival.^13^

In our centre, the high-risk criteria were defined based on published literature describing key determinants of morbidity and mortality in this patient population (**Figure 1**). Patients who met these criteria were initially treated with bPAB. If the risk factors were considered potentially modifiable, the most common being low birth weight, or prematurity patients were directed to a DN once adequate clinical optimization allowed reclassification closer to a standard-risk profile. If risk factors could not be mitigated in the early interstage period, patients mostly waited for a later comprehensive stage II palliation at 4-6 months of age.

Given that 60% of the patients in the current study had 3 or more risk factors prior to bPAB, with a median of 3 risk factors per patient, the survival rates, 95% at 30 days, 75% operative survival, and 55% at 1 year after the DN operation, were comparable to those observed in patients with fewer risk factors who underwent the Norwood operation as a primary surgical therapy. Also, the rate of therapeutic catheter-based interventions in 9 patients (45%) as well as the need for reoperation in 5 patients (25%) were similar to those reported in index studies.^7^^,^^8^^,^^15^

Matsunaga et al compared outcomes in a 3-centre study between DN performed at approximately 112 days of life and primary Norwood, utilizing patient selection independent of risk factors. While no differences in early surgical outcomes were observed, their study did not encompass a detailed perioperative course, Fontan completion rates, or long-term survival outcomes. Notably, the DN group demonstrated significantly reduced PA growth, persisting even 5 years post-Fontan.^16^ In our cohort, DN procedures performed within 4 weeks of life were associated with less PA interventions compared to the surgeries performed beyond 4 weeks, suggesting that if the PA bands were left in place around 4 weeks after the bPAB, the patients would require less PA reinterventions following the DN.

Hoashi et al reported intermediate outcomes in 45 patients who underwent the DN operation, 14 of whom had no identified risk factors. However, no detailed perioperative data were provided. While they demonstrated favourable intermediate survival rates of 84.5%, the Fontan completion rate was notably low. Additionally, systemic atrioventricular valve surgery was required in 18 patients (40.9%), and freedom from PA re-interventions was reported at only 27.1%.^17^ Similar studies have also reported higher interstage mortality rates. In a cohort of 24 HLHS patients with high-risk features, of whom 15 survived to DN, Guleserian et al described perioperative findings after bPAB comparable to ours in terms of immediate stabilization; however, detailed outcomes following the DN procedure were not provided.^18^ Our study aimed to shed light on the perioperative course as well as the causes of morbidity and mortality, thereby contributing to the characterization of this patient population.

In this study, we also observed a favourable early postoperative course concerning serum lactate peak levels, time to lactate normalization, vasoactive-inotropic scores, renal filtration, fluid balance, urine output, cNIRS values, and the use of opioid analgesics. Murtuza et al suggested that lactate clearance following the Norwood procedure could impact mortality and reported that 11 out of 12 neonates who failed to achieve sufficient lactate clearance within the first 24 hours did not survive.^19^ In our cohort, the median time to lactate normalization was significantly faster at 8 hours. Additionally, vasoactive-inotropic scores in our patient group were halved within the initial 48 hours.

Renal function at birth is characteristically low and improves progressively over the first 4 weeks of life.^20^^,^^21^ In patients undergoing the Norwood operation, renal function is frequently compromised by the surgical procedure itself. A peak in renal function deterioration was observed on the second postoperative day after Norwood procedure in the literature. Additionally, well-established risk factors for the Norwood procedure, such as low birth weight or low weight at the time of surgery, were also associated with poorer postoperative renal outcomes.^22^ In our patient cohort, both preoperative and postoperative renal function remained within normal limits and were not significantly affected by the surgery. This may be attributable to improved end-organ function facilitated by delaying the surgery beyond the initial weeks of life, ideally beyond the neonatal period, thereby allowing for organ maturation in this particularly vulnerable high-risk patient group.^13^^,^^23^

As anticipated, following the DN operation, patients exhibited higher reintubation rates, increased reliance on gastrostomy tubes, and prolonged hospital stays, reflecting their preoperative risk profile, including prematurity, low birth weight, and other extracardiac congenital anomalies. In addition, early extubation and more liberal protective reintubation strategies supported by robust non-invasive ventilation may have contributed to the higher reintubation rates. Similarly, feeding difficulties were anticipated given the degree of complexity of the patient group, yet survival in this high-risk group remained favourable, underscoring the importance of individualized supportive care. While numerous studies^16^^,^^24^ have suggested that DN is comparable to primary Norwood in terms of survival, our study uniquely demonstrated favourable 30-day survival rates and satisfactory in-hospital survival outcomes for this high-risk patient cohort. Although surgical and catheter-based reinterventions remained frequent, these were partially attributable to the patients’ underlying risk profiles. Notably, no patient required surgical intervention on the atrioventricular valves. Given the short median follow-up period of 1 year, only 10%-15% of eligible patients based on age had undergone Fontan palliation, and no patients underwent or were awaiting heart transplantation.

### Limitations

Limitations of this study include its retrospective, single-centre, non-randomized design and a small patient cohort with heterogeneous but significant preoperative risk profiles, limiting the feasibility of subgroup analyses. Furthermore, the study population represents a selected subgroup of patients who successfully underwent DN reconstruction following initial bPAB. Patients who died or underwent transplantation prior to DN although these numbers are low as presented, as well as patients transitioned to hybrid palliation, were excluded from the present analysis, introducing an inherent selection bias. Therefore, the reported outcomes should be interpreted as outcomes after successful bridging to DN rather than outcomes of the entire intended staged treatment strategy from the time of initial palliation.

For the freedom-from-reintervention analysis, death was treated as a censored event rather than a competing risk, given the small sample size and the limited number of observed events, which precluded robust estimation using competing-risk methodology and necessitated an exploratory time-to-event approach. Given the inherent nature of this very high-risk cohort, any direct comparison to patients undergoing primary neonatal Norwood procedures would introduce significant selection bias, as those patients are typically more haemodynamically stable and possess lower preoperative risk. Therefore, this study is intended to be descriptive rather than comparative in nature.

## CONCLUSION

Based on our centre’s evolving strategy and the encouraging early results presented here, we advocate for a “rapid two-stage Norwood” approach for all patients with HLHS.^13^ Our data demonstrated that bPAB^25^ prior to a DN operation may allow patients with universally recognized high-risk profiles to undergo a procedure at a later stage in life with improved anatomic and physiological features, achieving initial surgical outcomes comparable to those lacking risk factors. We, therefore, anticipate that adopting the RTSN approach—rather than performing a primary neonatal Norwood operation, during a period of rapidly evolving haemodynamics and endorgan function—may offer significant advantages.

## Supplementary Material

ivag218_Supplementary_Data

## Data Availability

The data underlying this article will be shared on reasonable request to the corresponding author.

## ABBREVIATIONS

BDG: bidirectional Glenn
bPAB: bilateral pulmonary artery banding
CI: confidence interval
CICU: cardiac intensive care unit
DN: delayed Norwood
DORV: double outlet right ventricle
ECMO: extracorporeal membrane oxygenation
HLHS: hypoplastic left heart syndrome
IQR: interquartile ranges
NIRS: near-infrared spectroscopy
PA: pulmonary artery
RTSN: rapid two-stage Norwood
VIS: vasoactive-inotropic support
