# Supplementary material for: Outcomes of Delayed Norwood Operation in High-Risk Patients—Time to Reconsider the Dogma of a Primary Norwood Operation?
Source: Interdiscip Cardiovasc Thorac Surg. 2026 Aug 28;41(9):ivag218. doi: 10.1093/icvts/ivag218 (PMC13541776; doi:10.1093/icvts/ivag218)
Supplement: Supplementary file 1 [file ivag218_supplementary_data.docx]

**Definitions**

Early (in-hospital) mortality was defined as death prior to hospital discharge or within 30 days after surgery.^1^ Late mortality was defined as all causes of death beyond 30 days after surgery and after hospital discharge. Prematurity was defined as a childbirth less than 37 gestational weeks of age. Low birth weight was defined as a birth weight 2.5 kilograms (kg) or less. Genetic abnormality was defined as those with a known genetic syndrome, or genetic lesions detected by chromosomal microarray analysis. A mean gradient across interatrial septal defect of more than 5 mmHg was considered to indicate restrictive interatrial communication. Acute kidney injury was defined based on the Pediatric modified Acute Kidney Injury Network Criteria^2^ as any of the following: an increase in serum creatinine to ≥1.5 times baseline within 24 hours, an increase in serum creatinine of ≥0.3 mg/dL within 48 hours, or a urine volume of <0.5 mL/kg/h for 6 hours. Cardiogenic shock was defined as hypotension, hyperlactatemia, and metabolic acidosis with pH<7.2. A normal serum lactate level is considered <2 mmol/L. Estimated glomerular filtration rate (GFR) was calculated using the creatinine-based “Bedside Schwartz” equation.^3^ Morphine equivalent dose was calculated using a conversion factor of 0.01:1 for fentanyl and 0.15:1 for hydromorphone.^4,5^ Patients who did not present to clinical follow-ups in two consecutive years were considered as lost to follow-up.

DN Surgery : The DN operation included standard steps of the primary Norwood, plus bilateral PA de-banding with intraoperative Hegar dilation or patch augmentation of branch PAs as needed, complete ductal stent removal, and either modified Blalock-Thomas-Taussig shunt or Sano modification placement under bicaval cannulation and selective cerebral perfusion at moderate hypothermia (26–28°C), with additional lower body perfusion per surgeon’s discretion.

Supplemental Table E1. Patient characteristics and perioperative outcomes of delayed Norwood after bilateral pulmonary artery banding (bPAB), stratified by timing of Norwood

|  | | | | | All delayed Norwood (n=20) | Early delayed Norwood; within 30 days of age (n=6) | Late delayed Norwood; after 30 days of age (n=14) | p-value |
| --- | --- | --- | --- | --- | --- | --- | --- | --- |
| Prematurity | | | | | 7 (35) | 1 (17) | 6 (43) | 0.35 |
| Weight at birth; kg | | | | | 2.8 (2.5-3.4) | 3.4 (3.1-3.5) | 2.6 (2-3) | 0.11 |
| Age at bPAB; days | | | | | 2.5 (2-4) | 2 (1-3) | 3 (2-4) | 0.21 |
| Reason of bPAB | Cardiogenic shock or early pulmonary over circulation | | | | 7 (35) | 3 (50) | 4 (29) | 0.61 |
|  | IUGR or low birth weight | | | | 11 (55) | 1 (17) | 10 (71) | **0.05** |
|  | Non-cardiac comorbidity | | | | 2 (10) | 2 (33) | 0 (0) | 0.08 |
| Ductal stent | | | | | 8 (40) | 1 (17) | 7 (50) | 0.32 |
| Age at ductal stent; days | | | | | 39 (28-67) | 7 (7-7) | 46 (32-70) | N/A |
| Ductal stent placement technique | | Percutaneous | | | 7 (35) | 1 (17) | 6 (43) | 0.35 |
|  |  | Hybrid | | | 1 (5) | 0 (0) | 1 (7) | 1 |
| Pre-Norwood complications | | In-stent stenosis | | | 3 (15) | 0 (0) | 3 (21) | 0.52 |
|  |  | Pericardial effusion | | | 2 (10) | 1 (17) | 1 (7) | 0.52 |
|  |  | Stent migration | | | 1 (5) | 0 (0) | 1 (7) | 1 |
|  |  | Subglottic stenosis | | | 2 (10) | 0 (0) | 2 (14) | 1 |
|  |  | ECMO | | | 1 (5) | 0 (0) | 1 (7) | 1 |
|  |  | NEC | | | 1 (5) | 0 (0) | 1 (7) | 1 |
| PA band maximum velocity; m/sec | | Right PA | | | 4 (3.7-4.2) | 4 (3.2-4.2) | 4 (3.8-4.3) | 0.28 |
|  |  | Left PA | | | 4.1 (3.8-4.4) | 3.8 (3.3-4.2) | 4.2 (3.9-4.5) | 0.20 |
| Age at delayed Norwood; days | | | | | 36 (29-79) | 21 (20-27) | 52 (35-99) | **<0.001** |
| PA Reconstruction during PA debanding | | Hegar dilatation | | | 20 (100) | 6 (100) | 14 (100) | N/A |
|  |  | Patch augmentation | | | 0 (0) | 0 (0) | 0 (0) |  |
| Postoperative ECMO | | | | | 11 (55) | 1 (17) | 10 (71) | **0.05** |
| e-CPR | | | | | 7 (35) | 0 (0) | 7 (50) | **0.05** |
| Early postoperative PS | | | | | 4 (20) | 1 (17) | 3 (21) | 1 |
| Postoperative CICU LOS; days | | | | | 57 (23-179) | 32 (21-44) | 80 (33-231) | 0.11 |
| Catheter reintervention for PS | | | | | 3 (15) | 0 (0) | 3 (21) | 0.52 |
| Reoperation for PS | | | | | 4 (20) | 0 (0) | 4 (29) | 0.27 |
| Catheter reintervention or reoperation for PS | | | | | 6 (30) | 0 (0) | 6 (43) | 0.11 |
| Lactate normalization time; hours | | | | | 15 (6-22) | 15 (6-28) | 15 (6-20) | 0.90 |
| Max. lactate within the first 48 h; mmol/L | | | | | 7 (4-10) | 6 (4-11) | 7 (4-10) | 0.86 |
| Urine output; mL/kg/h | | | | on POD 0 | 2.4 (1.5-3.8) | 3.3 (1.9-5) | 2.4 (1.4-3) | 0.23 |
|  |  |  |  | on POD 1 | 3.7 (2.9-5.3) | 5.2 (4.1-6.7) | 3.3 (2.6-4.7) | 0.25 |
| GFR; mL/min/1.73 m^2^ | | | | on POD 1 | 55 (49-61) | 49 (41-67) | 56 (52-61) | 0.48 |
|  |  |  |  | on POD 2 | 52 (42-67) | 49 (39-54) | 55 (42-68) | 0.65 |
| Vasoactive inotropic score | | | | 0-24 h | 14 (11-17) | 16 (14-20) | 14 (11-16) | 0.37 |
|  |  |  |  | 24-48 h | 12 (7-15) | 12 (6-15) | 12 (8-15) | 0.56 |
| cNIRS; % | | | | 0-24 h | 58 (51-61) | 56 (45-59) | 58 (53-61) | 0.32 |
|  |  |  |  | 24-48 h | 60 (55-65) | 60 (56-65) | 60 (54-65) | 0.4 |
| Fluid balance; mL | | | on POD 0 | | 336 (133-611) | 291 (203-578) | 426 (60-604) | 0.9 |
|  |  |  | on POD 1 | | -18 (-181- 46) | -17 (-196- 20) | -27 (-167- 55) | 0.88 |
| Post-op morphine equivalent dose; mg/kg | | | | | 46 (16-191) | 31 (14-51) | 55 (19-219) | 0.24 |
| In-hospital mortality | | | | | 5 (25) | 1 (17) | 4 (29) | 1 |
| Overall mortality | | | | | 9 (45) | 1 (17) | 8 (57) | 0.16 |

Values are represented as median (interquartile range Q1-Q3) or n (%). bPAB; bilateral pulmonary artery banding, CICU; cardiac intensive care unit, cNIRS; near infrared spectroscopic cerebral oxygen saturation, h; hours, ECMO; extracorporeal membrane oxygenation, e-CPR; extracorporeal cardiopulmonary resuscitation, GFR; estimated glomerular filtration rate, IUGR; intrauterine growth restriction, LOS; length of stay, mg/kg; milligrams per kilogram, mL; milliliter, mmol/L; millimoles per liter, m/sec; meters per second, N/A; not applicable, NEC; necrotizing enterocolitis, PA; pulmonary artery, POD; postoperative day, PS; pulmonary stenosis. * A normal serum lactate level is <2 mmol/L. Statistically significant p-values (p<0.05) are displayed in bold.

Supplemental Table E2. Data following bilateral pulmonary artery banding until delayed Norwood operation (n=20)

| Variables |  | |
| --- | --- | --- |
| Interval Between bPAB and DN; days | 33 (28-72) | |
| PGE Only | 12 (60) | |
| PGE followed by PDA Stent | 8 (40) | |
| Mechanical Ventilation; days | 4.5 (3-15) | |
| Highest VIS | 5 (3-9) | |
|  | **Post bPAB** | **Pre-Norwood** |
| Weight; kg | 2.8 (2.5-3.4) | 3.5 (3.2-3.9) |
| Head Circumference; cm | 34 (33-35) | 35 (35-36) |
| Length; cm | 47 (45-49) | 50 (48-53) |
| FR; mL/min/1.73 m^2^ | 41 (29-49) | 54 (39-86) |
| PO Feeding Only | 6 (30) | |
| PO + Tube Feeds | 3 (15) | |
| NPO Only | 6 (30) | |
| PO/Tube + Parenteral | 5 (25) | |
| NEC | 1 (5) | |

Values are represented as median (interquartile range Q1-Q3) or n (%). bPAB; bilateral pulmonary artery banding, DN; Delayed Norwood, GFR; glomerular filtration rate, IQR; interquartile range, NEC; necrotizing enterocolitis, NPO; nothing by mouth, PDA; patent ductus arteriosus, PO; per oral, PGE; prostaglandin E1, VIS; vasoactive inotropic score.

Supplemental Table E2. Early postoperative data in patients excluding patients having ECMO support.

| Variables | Delayed Norwood |
| --- | --- |
| Maximum VIS |  |
| POD 0 | 15 (13-18) |
| POD 1 | 14 (10-15) |
| POD 2 | 8 (8-9) |
| POD 3 | 8 (6-9) |
| Maximum lactate; mmol/L |  |
| POD 0 | 4.6 (3.8-9.1) |
| POD 1 | 2.1 (1.7-2.9) |
| POD 2 | 1.5 (1.4-2.3) |
| POD 3 | 1.2 (1-1.3) |
| Median cerebral NIRS; % |  |
| POD 0 | 58 (53-60) |
| POD 1 | 58 (54-63) |
| POD 2 | 53 (47-56) |
| POD 3 | 55 (50-57) |
| Urine output; ml/kg/h |  |
| POD 0 | 2.3 (1.6-4) |
| POD 1 | 4.8 (3.6-6.7) |
| POD 2 | 5.7 (4.5-7.3) |
| POD 3 | 4.8 (4.5-5.7) |
| GFR; 88 mL/min/1.73 m2 |  |
| POD 0 | 63 (55-77) |
| POD 1 | 58 (52-64) |
| POD 2 | 61 (48-70) |
| POD 3 | 64 (53-89) |
| Number of patients on ECMO |  |
| POD 0 | 5 |
| POD 1 | 8 |
| POD 2 | 8 |
| POD 3 | 8 |
| Discharge after ECMO; % | 75 |

Values are represented as median (interquartile range Q1-Q3). DN; delayed Norwood, GFR; glomerular filtration rate, NIRS; near infrared spectroscopic oxygen saturation, PN; primary Norwood, POD; postoperative day, VIS; vasoactive inotropic score. Values for patients on ECMO are excluded.

Supplemental Table E3. In-hospital mortality data of patients who underwent delayed Norwood (n=9)

| # patients | Primary diagnosis | Birth weight; kg | Time of delayed Norwood | Time of mortality; POD | Reason of mortality | Other risk factors |
| --- | --- | --- | --- | --- | --- | --- |
| 1 | MA, DORV | 2.9 | Within 4-6 weeks | 299 (following BDG) | Severe chronic lung disease, pulmonary hypertension, failing BDG, shock, sepsis |  |
| 2 | AA/MA | 2.6 | Within 4-6 weeks | 85 | LCOS, ECMO, multiorgan failure, sepsis |  |
| 3 | MA, DORV | 1.8 | After 6 weeks | 218 | e-CPR, severe global ischemic neurologic injury, severe tracheal stenosis | Heterotaxy,  TAPVR, IUGR, PPROM |
| 4 | AA/MS | 1.5 | After 6 weeks | 70 | 3 times e-CPR, LCOS, during MRI scanning unable to intubate | IUGR |
| 5 | AA/MS | 2.6 | Within 4-6 weeks | 19 | possible aspiration and e-CPR on POD 12, moderate TR, LCOS, Massive brain infarction | Genetic abnormality |

AA; aortic atresia, BDG; bidirectional Glenn, DORV; double outlet right ventricle, ECMO; extracorporeal membrane oxygenation, e-CPR; extracorporeal cardiopulmonary resuscitation, IUGR; intrauterine growth restriction, kg; kilogram, LCOS; low cardiac output syndrome, MA; mitral atresia, MS; mitral stenosis, POD; postoperative day, PPROM; preterm premature rupture of the membranes, TAPVR; total anomalous of pulmonary venous return, TR; tricuspid regurgitation.

Supplemental Table E4. Unplanned Reinterventions

|  | | Delayed Norwood n=20 |
| --- | --- | --- |
| Therapeutic catheter-based reintervention | Number of patients | 9 (45) |
|  | In-hospital reintervention | 6 (30) |
|  | Median time to catheter reintervention; days | 79 (45-97) |
|  | Arch balloon angioplasty and/or stenting | 3 (15) |
|  | Shunt balloon angioplasty and/or stenting | 5 (25) |
|  | Pulmonary artery balloon angioplasty and/or stenting | 3 (15) |
| Reoperation | Number of patients | 5 (20) |
|  | In-hospital reoperation | 5 (20) |
|  | Median time to reoperation; days | 2 (1-15) |
|  | Arch repair | 1 (5) |
|  | Shunt revision | 5 (25) |
|  | Pulmonary artery patch-plasty | 4 (20) |

Values are represented as median (interquartile range Q1-Q3) or n (%).

Supplemental Table E5. Data of following staged procedure

| Bidirectional Glenn Procedure | Delayed Norwood n=20 |
| --- | --- |
| Number of patients | 13 (65) |
| Age at BDG; months | 5.8 (4.9-6.1) |
| Concomitant pulmonary arterioplasty | 5 (25) |
| Concomitant aortoplasty | 2 (10) |
| Concomitant AV valve repair | 2 (10) |

Values are represented as median (interquartile range Q1-Q3) or n (%). AV valve; atrioventricular valve, BDG; bidirectional Glenn. Statistically significant p-values (p<0.05) are displayed in bold.

Supplementary References

1. Jacobs JP, Mavroudis C, Jacobs ML, et al. What is operative mortality? Defining death in a surgical registry database: a report of the STS Congenital Database Taskforce and the Joint EACTS-STS Congenital Database Committee. Ann Thorac Surg. 2006;81(5):1937-1941.

2. Mehta RL, Kellum JA, Shah SV, et al. Acute Kidney Injury Network: report of an initiative to improve outcomes in acute kidney injury. Crit Care. 2007;11(2):R31.

3. Schwartz GJ, Feld LG, Langford DJ. A simple estimate of glomerular filtration rate in full-term infants during the first year of life. J Pediatr. 1984;104(6):849-854.

4. Amirnovin R, Sanchez-Pinto LN, Okuhara C, et al. Implementation of a Risk-Stratified Opioid and Benzodiazepine Weaning Protocol in a Pediatric Cardiac ICU. Pediatr Crit Care Med. 2018;19(11):1024-1032.

5. Gordon DB, Stevenson KK, Griffie J, Muchka S, Rapp C, Ford-Roberts K. Opioid equianalgesic calculations. J Palliat Med. 1999;2(2):209-218.
